# Improvements in Upper Extremity Function Following Intensive Training Are Independent of Corticospinal Tract Organization in Children With Unilateral Spastic Cerebral Palsy: A Clinical Randomized Trial

**DOI:** 10.3389/fneur.2021.660780

**Published:** 2021-05-03

**Authors:** Kathleen M. Friel, Claudio L. Ferre, Marina Brandao, Hsing-Ching Kuo, Karen Chin, Ya-Ching Hung, Maxime T. Robert, Veronique H. Flamand, Ana Smorenburg, Yannick Bleyenheuft, Jason B. Carmel, Talita Campos, Andrew M. Gordon

**Affiliations:** ^1^Burke Neurological Institute, White Plains, NY, United States; ^2^Weill Cornell Medicine, New York, NY, United States; ^3^Teachers College, Columbia University, New York, NY, United States; ^4^Universidade Federal de Minas Gerais, Belo Horizonte, Brazil; ^5^Queens College, City University of New York, New York, NY, United States; ^6^Université Catholique de Louvain, Brussels, Belgium; ^7^Weinberg Family Cerebral Palsy Center, Columbia University Medical Center, New York, NY, United States

**Keywords:** hemiplegia, transcramial magnetic stimulation, Hand-Arm Bimanual Intensive Therapy (HABIT), rehabilitation, constraint-induced movement therapy (CIMT), brain reorganization, neuroplasicity, physical rehabilitation

## Abstract

**Background/Objectives:** Intensive training of the more affected upper extremity (UE) has been shown to be effective for children with unilateral spastic cerebral palsy (USCP). Two types of UE training have been particularly successful: Constraint-Induced Movement Therapy (CIMT) and Bimanual training. Reorganization of the corticospinal tract (CST) early during development often occurs in USCP. Prior studies have suggested that children with an ipsilateral CST controlling the affected UE may improve less following CIMT than children with a contralateral CST. We tested the hypothesis that improvements in UE function after intensive training depend on CST laterality.

**Study Participants and Setting:** Eighty-two children with USCP, age 5 years 10 months to 17 years, University laboratory setting.

**Materials/Methods:** Single-pulse transcranial magnetic stimulation (TMS) was used to determine each child's CST connectivity pattern. Children were stratified by age, sex, baseline hand function and CST connectivity pattern, and randomized to receive either CIMT or Bimanual training, each of which were provided in a day-camp setting (90 h). Hand function was tested before, immediately and 6 months after the intervention with the Jebsen-Taylor Test of Hand Function, the Assisting Hand Assessment, the Box and Block Test, and ABILHAND-Kids. The Canadian Occupational Performance Measure was used to track goal achievement and the Pediatric Evaluation of Disability Inventory was used to assess functioning in daily living activities at home.

**Results:** In contrast to our hypothesis, participants had statistically similar improvements for both CIMT and Bimanual training for all measures independent of their CST connectivity pattern (contralateral, ipsilateral, or bilateral) (*p* < 0.05 in all cases).

**Conclusions/Significance:** The efficacy of CIMT and Bimanual training is independent of CST connectivity pattern. Children with an ipsilateral CST, previously thought to be maladaptive, have the capacity to improve as well as children with a contralateral or bilateral CST following intensive CIMT or Bimanual training.

**Clinical Trial Registration:** www.ClinicalTrials.gov, identifier NCT02918890.

## Introduction

Unilateral spastic cerebral palsy (USCP) is characterized by sensorimotor deficits, particularly upper extremity (UE) impairments mainly on one side of the body. In the past decade, the evidence base for effective therapies has expanded considerably (1). The best available therapies for UE impairments in children with USCP involve intensive, skill-based motor training (2–7). Despite the general efficacy of these intensive interventions, there is considerable individual variability in responsiveness (8–10). The high costs and time associated with intensive therapy highlight the need for a greater understanding of neurophysiological mechanisms that may mediate functional recovery and can be targeted to optimize interventions.

One potential key determinant to how children respond to UE therapies is developmental adaptation of the motor system following early brain injury. The principal pathway for control of skilled UE movements is the corticospinal tract (CST) (11). During typical development, early bilateral projections of the CST are pruned leading to a predominantly contralateral system (12). In the context of a perinatal injury, there is often a loss of direct projections of the CST from the injured motor cortex to contralateral spinal cord motor circuits (13). Depending on the timing and size of injury, there may be an aberrant reorganization of the motor system in which the uncrossed (i.e., ipsilateral) projections from the non-lesioned hemisphere retain control of the affected hand (14). These ipsilateral connections, found in more than half of children with USCP, have been considered to be “maladaptive” (15, 16), and those who retain contralateral projections have better hand function than those who do not [e.g., (10, 17–19)]. However, there is growing evidence that ipsilateral pathways have the capacity to support substantial motor function (20, 21). The ipsilateral motor map can contain an abundance of distal UE representations and are plastic (10, 21, 22). In fact, greater relative overlap of the affected and less affected hand representation within the contralesional hemisphere has been shown to be associated with better hand function (23). This suggests that overlapping representations might be adaptively “yoked,” with cortical control of the child's less affected hand supporting that of the affected hand.

Two types of training have been shown to be among the most efficacious approaches to improving UE function (1). Constraint-induced movement therapy involves restraint of the less affected UE and intensive unimanual practice with the more affected UE [e.g., (24)]. Bimanual training involves provision of tasks that necessitate or instruct use of both UE with the more affected UE generally used as an assist (25). Both approaches have been found to result in similar efficacy for most clinical outcomes [e.g., (2, 26–30)]. However, the heterogeneity in children with UCSP described above raises important questions about the relation between CST pathway reorganization and treatment outcome. Studies of CIMT have suggested that children with ipsilateral control of the affected UE show markedly reduced improvements in movement speed (16) and cortical excitability (31) compared to children with a contralateral CST, possibly due to the absence of typical interhemispheric inhibition and/or a potential hemispheric dissociation of affected hand representations in the primary motor cortex and somatosensory cortex in these children (16, 31–33). In contrast, we have shown that improvements in skill and increases in motor maps occur independently of CST laterality following bimanual training (10, 21). Thus, CST laterality (especially ipsilateral reorganization) may predict outcomes, depending on whether the training is unimanual or bimanual. Despite the growing awareness of CST laterality as a potential biomarker of recovery, its relation to CIMT and bimanual training, two approaches with high levels of evidence for improving motor function in children with USCP (1), has not been formally tested in a large-sample prospective trial.

In the present study we tested the hypothesis that improvements in UE function following either CIMT or bimanual training depend on CST laterality and type of training (unimanual vs. bimanual) in children with USCP. In particular, we predict that whereas children with a maintained contralateral CST will respond equally to CIMT and bimanual training, children with ipsilateral CST laterality will be less responsive to CIMT than bimanual training. We tested our hypothesis in a randomized clinical trial in which children were randomized to receive either 90 h of CIMT or Hand-Arm Bimanual Intensive Training (HABIT).

## Methods

All study procedures were approved by the Institutional Review Boards of Teachers College, Columbia University, where the treatments were conducted, the Burke Neurological Institute, where TMS evaluations were performed, and Weill Cornell Medicine, where magnetic resonance imaging (MRI) was performed. Children and their caregivers provided written informed assent and consent.

### Participants

Demographics and clinical characteristics of participants are provided in Table 1. Participants were recruited from clinics in the NYC area, our Web site (http://www.tc.edu/centers/cit/), ClinicalTrials.gov (NCT02918890), and online support groups. All participants were randomized (see below) to receive Hand-Arm Bimanual Intensive Training (HABIT) or Constraint-Induced Movement Therapy (CIMT) program, delivered in a day camp model (6 h/day, 15 days). Six cohorts participated in the intervention delivered in a summer day camp setting. The inclusion criteria for were: (1) age 5.5–17 years, (2) diagnosed with USCP, (3) capable of participating in a 15 day, 6 h/day camp while separated from caregiver(s), (4) capable of following directions regarding hand use and testing, (5) capable of communicating needs, (6) mainstreamed in age-appropriate school classroom, and (7) able to lift the more affected arm 15 cm above a table surface and grasp light objects. The exclusion criteria were: (1) unwillingness to comply with instructions or other behavioral issues making delivery of an intensive therapy infeasible, (2) health problems unassociated with hemiplegia, (3) visual impairment that could interfere with participation, (4) orthopedic surgery on the more affected hand within 1 year, (5) presence of metallic objects in the body, and (6) botulinum toxin in the more affected upper extremity within the past 6 months or intended treatment within the study period, (7) seizures after the age of 2 years, (8) family history of seizure disorders, (9) current medication use to lower the seizure threshold, (10) claustrophobia, or (11) pregnancy. Sample size calculations were based on the results of the Assisting Hand Assessment (AHA) and Jebsen-Taylor Test of Hand Function (JTTHF) outcomes of a prior CIMT/HABIT RCT (2) and pilot data. The difference in effect size of improvement in hand function was estimated to be 0.35 (difference change JTTHF = 102 s, sd = 120, AHA change 7 logits, sd = 10), alpha = 0.05, beta = 0.8 and 20% potential dropout. The analysis yielded 82 children.

**Table 1:** Included participant characteristics.

	**CIMT group**	**HABIT group**
*N*	40	39
Mean age in years, months (SD)	9.4 (2.10)	9,7 (3,5)
**Sex**		
Male	27	21
Female	13	18
**Affected hemisphere**		
Right	19	14
Left	21	25
**Lesion type**		
Middle cerebral artery	10	13
Periventricular	25	23
Malformation	2	1
Unknown	3	2
**Corticospinal tract laterality**		
Contralateral	7	3
Bilateral	7	6
Ipsilateral	26	30
**MACS**		
I	9	12
II	25	21
III	6	6
Baseline AHA, mean (SD) (95% CI), logits	56.7 (20.7) (50.1, 63.3)	55.2 (8.7) (52.4, 58.1)
Baseline JTTHF, more-affected, mean (SD) (95% CI), sec	405.1 (306.8) (306.9, 503.2)	402.5 (296.5) (306.4, 498.7)
Baseline JTTHF, less-affected, mean (SD) (95% CI), sec	56.7 (20.7) (39.1, 74.3)	63.8 (40.0) (43.8, 83.9)
Baseline COPM Perf, mean (SD) (95% CI), score	2.9 (1.1) (2.5, 3.2)	3.0 (1.4) (2.5, 3.4)
Baseline COPM Sat, mean (SD) (95% CI), score	3.1 (1.6) (2.6, 3.6)	3.3 (1.8) (2.7, 3.9)
Baseline BBT, more-affected, mean (SD) (95% CI), sec	18.2 (10.1) (15.0, 21.5)	16.6 (9.8) (13.5, 23.0)
Baseline BBT, less-affected, mean (SD) (95% CI), sec	42.9 (12.7) (29.6, 56.1)	41.1 (12.8) (28.2, 54.0)
Baseline PEDI-FS, mean (SD) (95% CI), score	63.8 (6.1) (61.9, 65.8)	63.2 (7.0) (61.0, 65.5)
Baseline PEDI-CA, mean (SD) (95% CI), score	33.8 (5.4) (32.0, 35.5)	33.2 (5.7) (31.9, 35.6)
Baseline ABILHAND-Kids, mean (SD) (95% CI), score	1.9 (1.6) (1.4, 2.4)	1.7 (1.1) (1.4, 2.1)

*MACS, Manual Ability Classification System; AHA, Assisting Hand Assessment; JTTHF, Jebsen-Taylor Test of Hand Function; COPM, Canadian Occupational Performance Measure; BBT, Box and Block Test; PEDI, Pediatric Evaluation of Disability Inventory (FS, Functional skills subscale; CA, Caregiver assistance in self-care subscale)*.

### Randomization

Before randomization, children completed all baseline outcome measures. Block randomization was implemented for each cohort of participants (8–18 children). Each cohort was stratified by age, sex, baseline hand function, and CST connectivity pattern as closely as possible, then randomized offsite using concealed allocation to receive either CIMT or HABIT.

### Interventions

#### General Intervention Procedures

Children participated in an intensive hand training intervention using either one (CIMT) or both (HABIT) hands. Children attended for 6 h/day over 15 days (90 h). During the intervention, children were paired with a trained interventionist, with an interventionist:child ratio of at least 1:1. The interventionists included physical and occupational therapists, graduate students in kinesiology/neuroscience, speech pathology, and psychology, and undergraduates. Interventionists were supervised by experienced PT/OTs who enforced protocol fidelity, and both the interventionists and supervisors were blinded to CST connectivity patterns. Prior to the intervention, a training session was administered by the supervisors and standardized based on the established manual of procedures for CIMT and HABIT. Fidelity was reinforced by supervisors during the day camp and during daily post-camp meetings. Participants receiving CIMT and HABIT were located in separate rooms. Each room was supervised by additional experienced PTs/OTs, who modeled and ensured uniformity of treatment. Each day, interventionists had team meetings to discuss the progress and needs of each child.

Participants worked one-on-one with their interventionist or in groups (while still paired with individual interventionists). Interventionists were matched with children prior to randomization considering the child's age, sex, and interests. Emphasis was placed on making the experience enjoyable. Activities were divided into whole and part task practice. Whole task practice involved sequencing successive movements within the context of activities (e.g., games, arts and crafts, goal training). The activities were performed continuously for at least 15–20 min. Targeted movements and spatial and temporal coordination were practiced within the context of completing the task. Part task practice (analogous to “shaping” in the psychology literature) (25, 34) involved breaking down motor skills into smaller components and reinforcement of successive approximations of the desired behavior (e.g., card turning to promote forearm supination) while increasing repetitions and progressing skill requirements. This approach also served to increase treatment intensity by requiring as many repetitions as possible over repeated 30-s intervals (typically a minimum of 5 intervals).

Task difficulty was graded by varying the spatial/temporal constraints or by providing tasks that required progressive skilled use as task performance improved. The difficulty was increased when the participant was successful on 7 of 10 repetitions. Task performance was recorded, and positive reinforcement and task- and age-specific knowledge of results were provided for encouragement (35).

#### Constraint-Induced Movement Therapy

CIMT was modified to be child-focused (2, 34). The restraint consisted of a cotton sling fastened to the child's trunk with the distal end enclosed to prevent using the less affected arm or hand as an assist. The sling was continuously worn throughout this intervention except when a break was requested (<0.5 h per 6 h session).

##### Task Selection

To engage the child in the intervention and to maintain engagement, we established a list of fine motor and manipulative gross motor activities that elicit movement behaviors of interest and included a battery of age-appropriate, unimanual functional and play activities. Interventionists selected tasks based on which train to the targeted hand impairments and the child's interest. Task difficulty was progressed as children improved by requiring greater speed, accuracy, or movement repetition.

#### Hand-Arm Bimanual Intensive Therapy

##### Task Selection

We previously identified age-appropriate fine and gross motor activities that require use of both hands (2, 25). Activities were chosen by taking into consideration the role of the more affected UE increasing in complexity from passive assist to active manipulator. While task demands were graded to allow success, children were typically asked to use the more affected UE in the same manner as that of the non-dominant limb of a typically developing child. Directions were provided to the child before the start of each task in order to avoid use of compensatory strategies. These directions specified how each hand will be used during the activity, although choice was often provided to keep the approach child-centered (e.g., use the more affected hand to roll the dice or move a board piece during a game) (2, 25).

#### Outcome Measures

We chose several measures of hand function to capture different aspects of manual ability. Assessments were administered by an experienced physical or occupational therapist who was blinded to the treatment allocation and CST connectivity of each child. The Assisting Hand Assessment (AHA, version 4.4) measured bimanual hand use. The Jebsen-Taylor Test of Hand Function (JTTHF) measured unimanual dexterity of the affected hand. The AHA and JTTHF were pre-determined primary outcome measures.

Several secondary outcome measures were also used. The Box and Blocks Test (BBT) measured unimanual dexterity. The Canadian Occupational Performance Measure (COPM) measured caregiver perceptions of a child's performance of functional goals, and satisfaction with how well the child can perform the goal. The ABILHAND-Kids is a parent-report of child's manual ability. The Pediatric Evaluation of Disability Inventory (PEDI) was used to measure functioning in the home environment (i.e., self-care domain). All measures were administered immediately before treatment (pre-test), within 2 days after (post-test), and 6 months after treatment (followup).

#### Bimanual Hand Function

##### AHA

The AHA is a validated test for measuring bimanual hand use in children with UE impairments. The AHA measures the use of the more affected hand in bimanual activities during a play-like testing session (36). Sessions were videotaped and scored off-site by a blinded evaluator. The AHA has excellent validity, reliability (0.97–0.99) and responsiveness to change (37). The AHA units were used for the analysis. The smallest detectable difference (SDD) for AHA is an improvement of at least 5 units (38).

#### Unimanual Dexterity

##### JTTHF

The JTTHF measures the time taken to complete six unimanual tasks, which include flipping cards, moving small objects, and lifting cans (39, 40). The total score is the amount of time taken to complete all tasks. The test was performed on both the more affected and less affected hands. The JTTHF is well-validated and has excellent reliability (40, 41).

##### BBT

The BBT measures how many blocks (2.5 cm^3^) an individual can move from one box, over a barrier, to an adjacent box in 1 min (42). Both hands were tested. The BBT is valid and reliable for children with CP (41).

#### Hand Use in Daily Functioning

##### COPM

The COPM is a structured interview in which the individuals are asked to identify up to five functional goals (43). In this study, parents reported their child's functional goals. Parents rated how well children perform each goal (COPM-Performance), and how satisfied they were with the child's performance (COPM-Satisfaction). The same caregiver was interviewed before and after the intervention. A change of 2 or more points in each scale of COPM is considered a minimum clinically important difference (MCID) (43). The COPM has been validated for parents of children with disabilities (44).

##### ABILHAND-Kids

The questionnaire measures the ability of a child to perform specific 21 daily tasks which require hand use, according to the parent's perspective (45). It has been validated for children with CP over the age of 6 and it is a reliable test (45, 46).

##### PEDI

Caregivers were interviewed to assess children's daily functioning using the PEDI, a valid/reliable test (47) focusing on child's functioning in daily living activities at home (48). Children's functional skills (PEDI-FS) and caregiver assistance (PEDI-CA) in self-care were assessed.

### Determination of CST Laterality

We determined CST laterality in two ways. (1) TMS (primary approach): We determined which hemisphere evokes muscle activation of the affected hand when TMS is applied to the primary motor cortex (M1); (2) DTI (secondary approach): We used DTI to visualize the affected CST only in children whose CST laterality could not be determined with TMS. We have shown that DTI is an accurate surrogate measure of CST laterality (49).

### Transcranial Magnetic Stimulation

Single-pulse transcranial magnetic stimulation (TMS) was used to determine which hemisphere's M1 controlled movement of the child's more affected hand. We recorded EMG from the first dorsal interosseous (FDI) muscle in both hands. Skin was cleaned with rubbing alcohol and a mild abrasive (NuPrep, Weaver and Company, Aurora, CO). Electrodes were placed on the FDI muscle belly. Reference electrodes were placed on the muscle tendon, and a ground electrode was placed on the wrist styloid process. EMG was recorded with Neuroconn hardware and software (Neuroprax, Germany). The Neuroconn received a trigger input from the TMS stimulator, such that the relative timing of an EMG response to a stimulus could be measured and visualized. The EMG response to TMS is a motor evoked potential (MEP).

We identified the spot at which a single TMS pulse evoked the strongest MEP in the affected FDI muscle (the motor “hotspot”). To identify the motor hotspot, single TMS pulses were delivered to the child's scalp, starting ~4 cm from midline above the ear. The initial TMS stimulus intensity was 50% stimulator output. If an MEP was not found, the coil position was moved in 1 cm increments to stimulate the scalp above motor cortex on both hemispheres. Stimulus intensity was increased in 2–5% increments until an MEP was found (50). We stimulated up to 80% stimulator output, because higher stimulation can be painful to participants. If we were unable to find an MEP in the motor strip, we stimulated at 80% stimulator output at 50 points across frontal and parietal cortices in one hemisphere. If no MEP was found at any of these sites, we classified that hemisphere as having no direct control of the movement of either upper extremity.

After the motor hotspot was found, the resting motor threshold (rMT) was determined. The rMT was defined as the minimum stimulus intensity needed to evoke an MEP in the affected FDI in 6 of 10 trials. Stimuli were delivered at a frequency <0.1 Hz. If an MEP was found after 6 of 10 pulses, the stimulus intensity was lowered 2% until an MEP was no longer found in 6 of 10 trials.

We then placed a circular grid over the hemisphere, centered over the hotspot, using Brainsight. The grid had a 10 cm diameter, with five concentric rings, each gridpoint placed 1 cm apart. The grid was centered over the hotspot for that hemisphere. Although there was always one maximally responsive hotspot, by stimulating each point of the grid, we captured all other responsive regions in motor cortex. We stimulated each site 1–3 times (2–3 times if a response was found) at 110% the participant's rMT. By stimulating at 110% rMT, we thoroughly searched for all motor cortex representations of the upper extremities. Responses, as described below, were sites at which a TMS stimulus evoked an MEP 50 μV or larger. As described in detail elsewhere, we calculated the ratio between the number of responses in the more affected FDI obtained from the lesioned and contralesional hemispheres (10, 51, 52). This ratio is the laterality index (LI).

Participants were categorized as having a contralateral CST connectivity pattern if the LI was between 0.9 and 1; i.e., 90–100% of the responses in the more affected hand come from the lesioned hemisphere. Participants were categorized as having an ipsilateral CST connectivity pattern if the LI was between 0 and 0.1; i.e., 0–10% of the responses in the affected hand come from the lesioned hemisphere. Participants were categorized as having a bilateral CST connectivity pattern if the LI was between 0.1 and 0.9.

### Transcranial Magnetic Stimulation Analysis

The latency and peak-to-peak amplitude of each TMS pulse was measured using a suite of custom-written MATLAB (Mathworks, Waltham, MA) scripts. If the latency of the MEP was longer than 40 ms after the TMS pulse, that trial was excluded from analysis. Additionally, if high levels (>100 μV) of background EMG activity were seen before the MEP, the trial was excluded from analysis.

### Magnetic Resonance Imaging

Each child received a structural MR scan (MP-RAGE, 3D, T1-weighted) and diffusion tensor imaging scan, without sedation prior to participation. The structural MRI was used to co-register TMS stimulation targets with specific brain landmarks, using a frameless stereotaxic neuronavigation system (Brainsight Frameless, Rogue Research, Montreal, QC, Canada). For TMS localization, there is normal variability in brain topography relative to scalp landmarks. MR scans were done on a Siemens Prisma MRI Scanner (Malvern, PA) in the Citigroup Biomedical Imaging Center (CBIC). Structural scans (165 slices) were taken at a resolution of 256 × 256 px. The structural MRI was used to identify the lesion type and extent, as well as to localize the TMS coil (i.e., neuronavigation). The DTI scan was done during the same session as the structural MRI. For DTI, a 65-direction protocol was used, 75 slices per direction at a resolution of 112 × 112 px each.

### Diffusion Tensor Imaging Analysis

DT images for participants whose CST laterality could not be determined with TMS due to excessively high threshold or safety reasons were imported into DTI Studio (Johns Hopkins University) software for processing and analyses. This has been shown to be a reliable surrogate for TMS in determining CST laterality (22, 49). Image series for each participant were screened for movement artifact, and slices showing artifact were removed. Since obtained images using 65 gradients and performed duplicate scans, up to 30% of slices may be removed without compromising feasibility of tract reconstruction. Using DTI Studio, we placed region of interest seeds in the affected motor cortex and cerebral peduncle, and later in the unaffected motor cortex and cerebral peduncle. We used tractography to find tracts that passed through both ROIs. We categorized each CST as present or absent. If there was a present CST on the affected side, the child was categorized as having a contralateral CST. If there was not a CST present on the affected side, and a CST present on the other side, the child was categorized as having an ipsilateral CST (49). Note that, with this approach, “bilateral” CST connectivity cannot be detected.

### Statistical Analysis

This was an intention-to-treat study. If a child missed their 6 month assessment, we imputed their missing data based on the average change data for other participants in their subgroup. Statistical analyses were performed using SPSS (IBM, version 26). A treatment (CIMT, HABIT) × CST connectivity pattern (contralateral, ipsilateral, bilateral) × test session (pre-, post-, 6 month) ANOVA with repeated measures on test session was performed on all measures and Newman-Keuls *post-hoc* tests were performed where appropriate. Regression analyses were done to determine predictors of outcomes. For children with a bilateral CST, correlations were done between the LI and changes in outcome measures. Statistical significance was considered at the *p* < 0.05 level.

## Results

### Patient Flow

**Figure 1:**
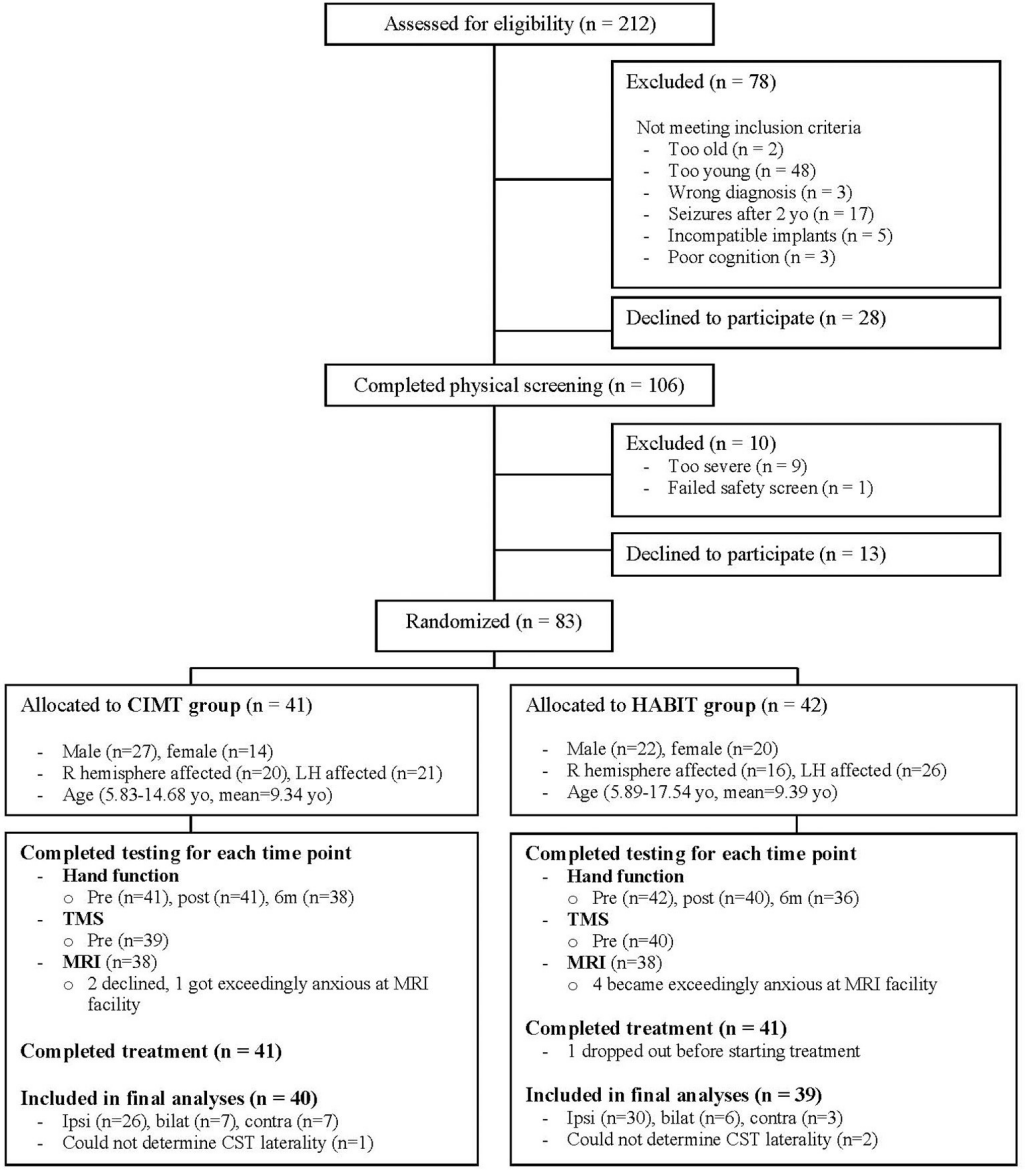
CONSORT flow diagram showing progress through the stages of the study, including flow of participants, withdrawals, and inclusion in analyses. A total of 212 individuals were screened by phone or e-mail. Of these 78 children did not meet the study criteria and 28 declined participation. The remaining 106 children potentially met the study criteria and were invited to undergo physical screening. Ten children were excluded and 13 who qualified declined to participate. The remaining 83 children were stratified by age, sex, baseline hand function and CST connectivity pattern, and randomized to receive either CIMT or HABIT. One child in the HABIT group dropped out before starting treatment, and 41 children in each group completed the intended treatments. We were unable to complete TMS or DTI on 3 participants due to exceedingly high thresholds or safety concerns, so only data of the 79 participants with CST determination were included (39 for CIMT, 40 for HABIT).

Patient flow is shown in the CONSORT diagram (Figure 1, see legend for details). During recruitment, 212 individuals were screened. Ultimately, 83 qualified individuals agreed to participate and were randomized to CIMT or HABIT. One child was randomized to HABIT after pre-test, but chose not to participate on the first day of treatment due to social anxiety. Thus, 82 participants (41 per group) completed the treatment, although we were unable to complete TMS or DTI on 3 participants so only data of the 79 participants with CST determination were included (39 for CIMT, 40 for HABIT). All other children completed the intervention, along with pre- and immediate post-intervention assessments, but 7 children missed the 6 month follow up assessments (CIMT *n* = 3, HABIT *n* = 4) and their data points were imputed. The results were the same whether or not data for these children were included. We were unable to determine the CST connectivity pattern for 3 children. The results were the same whether or not data for these children were included as well. Lesion type was missing for 5 children who declined MRIs. These 5 children were excluded from analyses of lesion type. Table 1 describes participant characteristics. There were no significant treatment group differences in baseline scores for any measure.

### Adverse Events

Five children had adverse events. One child had a seizure 9 days after completing the immediate post-test. The event took place immediately after an overseas flight without sleep and after the child fell from bed. Two children had seizures (one suspected and one confirmed) shortly before the 6-month follow up. One child broke the more affected UE between the immediate and 6-month follow up. Six-month follow up evaluations were not conducted on these 4 participants. A fifth child fell and required stitches in the head within a month of the 6-month follow up. This child completed clinical testing for the 6-month follow up. None of the events were deemed to be study-related.

### Bimanual Hand Function

**Figure 2:**
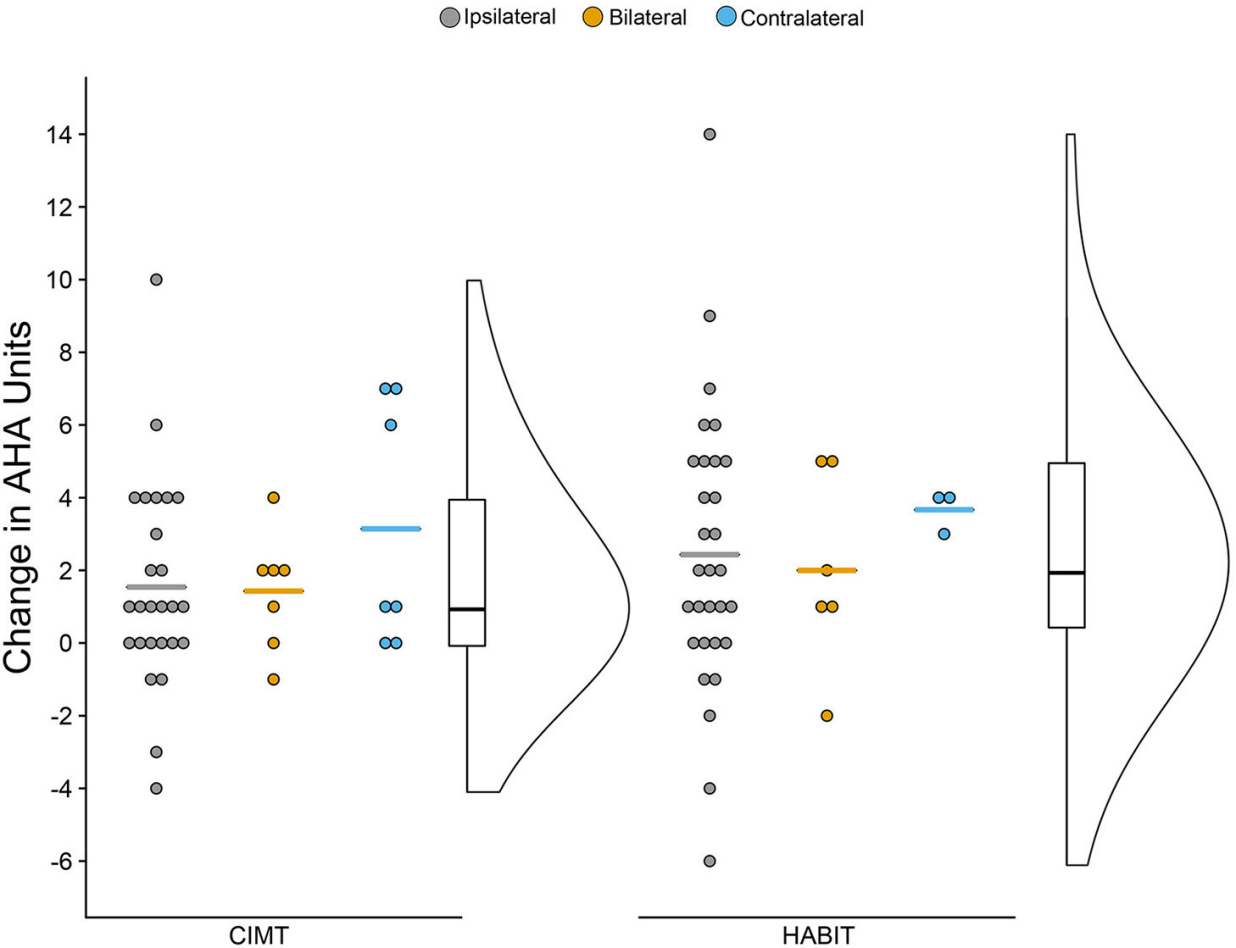
Raincloud Plot of Changes (Pre-test to Post-test) in AHA by Therapy Group and CST Connectivity. Dots represent raw change scores for individual children (positive scores = improvements). Horizontal colored lines represent mean of CST group. Boxplots represent median and quartiles of therapy group data. Curve represents probability distribution of therapy group data.

Figure 2 shows the change scores for the AHA as a function of treatment and CST organization (mean scores can be seen in Table 2). As seen in the figure, overall there was improvement across both treatments and CST organization patterns, but there were variations within each group. There was a significant increase of 1.8 and 2.4 AHA-units for CIMT and HABIT, respectively, across test sessions {[*F*_(2, 72)_ = 14.91, *p* < 0.001, partial eta^2^ = 0.11], Table 2 and Figure 2}. Five participants in the CIMT group and 11 in the HABIT group reached the SDD. Newman-Keuls *post-hoc* tests revealed a significant improvement between the pre-test and immediate post-test that was maintained at 6 months. There were no interactions between intervention group and test session or intervention group, CST connectivity pattern and test session (*p* > 0.05).

**Table 2:** Outcome measures.

	**Pre-test (95% CI)**	**Post-test (95% CI)**	**6 m follow-up (95% CI)**
**CIMT**			
**AHA (0–100 units)**			
Ipsilateral CST	52.5 (48.5, 56.6)	54 (50.2, 57.8)	54.4 (50.3, 58.4)
Bilateral CST	62.3 (57.1, 67.5)	63.7 (57.6, 69.8)	64.1 (57.8, 70.5)
Contralateral CST	55.4 (50.4, 60.5)	58.6 (52.6, 64.5)	58.1 (52.4, 63.9)
**JTTHF, more-affected (seconds)**			
Ipsilateral CST	479.5 (358.5, 600.5)	354.1 (254.5, 453.7)	342.4 (233, 451.9)
Bilateral CST	265.6 (46.6, 484.6)	175.9 (−28.8, 380.7)	213.3 (13.6, 413)
Contralateral CST	323.6 (89.9, 557.3)	224 (47.4, 400.7)	266.1 (109.5, 422.7)
**JTTHF, less-affected (seconds)**			
Ipsilateral CST	61.7 (53.9, 69.6)	58.3 (49.8, 66.8)	53.3 (45.7, 60.8)
Bilateral CST	47.3 (38, 56.5)	47.5 (34.8, 60.1)	49.1 (41.4, 56.8)
Contralateral CST	49.4 (35.2, 63.5)	43.3 (34.4, 52.2)	46.1 (33, 59.1)
**COPM Performance (0–10 rank)**			
Ipsilateral CST	2.9 (2.5, 3.3)	5.7 (5, 6.4)	6 (5.3, 6.7)
Bilateral CST	3.2 (2.5, 3.9)	6.3 (5.3, 7.4)	6.6 (5.7, 7.4)
Contralateral CST	2.5 (1.2, 3.8)	4.8 (3.3, 6.4)	5.3 (4.6, 6.1)
**COPM Satisfaction (0–10 rank)**			
Ipsilateral CST	3.2 (2.6, 3.8)	6.4 (5.5, 7.3)	6.6 (5.9, 7.3)
Bilateral CST	3 (1.8, 4.3)	7.2 (6, 8.4)	7.3 (6.5, 8.2)
Contralateral CST	2.9 (1.9, 4)	5.7 (3.8, 7.7)	6.3 (5, 7.5)
**BBT, more-affected (*****n*** **of blocks)**			
Ipsilateral CST	16.6 (12.1, 21.1)	19.2 (13.9, 24.4)	19.7 (14.6, 24.8)
Bilateral CST	21.4 (15, 27.9)	27 (20.5, 33.5)	24.7 (17.4, 31.9)
Contralateral CST	18.9 (14.7, 23.1)	25.6 (21, 30.1)	23.4 (18.7, 28)
**BBT, less-affected (seconds)**			
Ipsilateral CST	42.2 (36.9, 47.6)	46.6 (40.5, 52.7)	46.9 (41.5, 52.3)
Bilateral CST	44.9 (35, 54.8)	48.9 (37.6, 60.1)	50.5 (38.8, 62.3)
Contralateral CST	42.9 (38.4, 47.3)	48.1 (41.6, 54.7)	48 (44, 52)
**PEDI functional skills**			
Ipsilateral CST	63.4 (61.1, 65.6)	66.5 (64.7, 68.3)	67.7 (66.1, 69.3)
Bilateral CST	64.9 (58.8, 70.9)	69.1 (65.8, 72.5)	69.5 (66.3, 72.7)
Contralateral CST	64.3 (61.3, 67.2)	65.3 (62.5, 68)	66 (63.4, 68.6)
**PEDI caregiver assistance**			
Ipsilateral CST	33.7 (31.4, 36)	35.6 (33.8, 37.4)	37.4 (35.7, 39.1)
Bilateral CST	35 (31.4, 38.6)	35.9 (32, 39.8)	36.9 (32.3, 41.5)
Contralateral CST	33.9 (30.7, 37)	35.1 (32.3, 37.9)	35.9 (35.1, 36.6)
**ABILHAND-Kids**			
Ipsilateral CST	1.6 (1, 2.3)	2.3 (1.7, 3)	2.7 (2.1, 3.2)
Bilateral CST	2.2 (1, 3.4)	2.5 (1.2, 3.8)	2.8 (1.4, 4.3)
Contralateral CST	2.6 (1.8, 3.3)	2.4 (1.6, 3.1)	2.3 (1.2, 3.4)
**HABIT**			
**AHA (0–100 units)**			
Ipsilateral CST	54.8 (51.4, 58.1)	57.2 (54.1, 60.3)	56.5 (53.4, 59.5)
Bilateral CST	54.5 (48.1, 60.9)	56.5 (49.4, 63.6)	56.7 (50.5, 62.8)
Contralateral CST	61.3 (57.9, 64.8)	65 (57.9, 72.1)	66.3 (62.9, 69.8)
**JTTHF, more-affected (seconds)**			
Ipsilateral CST	435.7 (331.2, 540.2)	378.6 (281.3, 476)	380.2 (274.2, 486.2)
Bilateral CST	377.3 (122.4, 632.1)	316.8 (90.8, 542.8)	323.8 (152, 495.5)
Contralateral CST	121.3 (−183.9, 426.5)	98.9 (−170.5, 368.4)	146 (−152, 444)
**JTTHF, less-affected (seconds)**			
Ipsilateral CST	70.6 (65.6, 75.5)	64.9 (58.7, 71.1)	55.4 (50.5, 60.3)
Bilateral CST	41.5 (−23.1, 106.2)	42.6 (−13.3, 98.5)	42.1 (21.2, 63)
Contralateral CST	41.9 (12.1, 71.7)	47 (35.2, 58.8)	44.2 (12.5, 75.8)
**COPM performance (0–10 rank)**			
Ipsilateral CST	2.8 (2.3, 3.4)	6.4 (5.8, 7)	6.1 (5.5, 6.6)
Bilateral CST	3.6 (2.9, 4.2)	6.5 (5, 8.1)	6.5 (4.9, 8)
Contralateral CST	3.5 (3.2, 3.7)	5.9 (4.6, 7.1)	6.9 (5.5, 8.4)
**COPM satisfaction (0–10 rank)**			
Ipsilateral CST	3.3 (2.6, 4)	7 (6.4, 7.7)	6.5 (5.8, 7.1)
Bilateral CST	2.7 (2.1, 3.3)	6.8 (5.5, 8.1)	6.8 (5.5, 8.1)
Contralateral CST	4.4 (2.1, 6.7)	6.1 (3.7, 8.5)	7.7 (5.9, 9.5)
**BBT, more-affected (*****n*** **of blocks)**			
Ipsilateral CST	16.2 (12.5, 19.9)	19.2 (15.6, 22.8)	19.3 (15.6, 23)
Bilateral CST	14 (7.9, 20.1)	18.7 (10, 27.4)	18.2 (9.5, 26.9)
Contralateral CST	26 (19.2, 32.8)	27 (20.6, 33.4)	31.3 (19.7, 42.9)
**BBT, less-affected (seconds)**			
Ipsilateral CST	39.9 (35.2, 44.6)	43.8 (39.8, 47.8)	46.7 (42.1, 51.3)
Bilateral CST	46.3 (36.5, 56.1)	49.2 (36.9, 61.4)	49.4 (37.5, 61.3)
Contralateral CST	43 (30.3, 55.7)	51.3 (44.7, 58)	56 (41.2, 70.8)
**PEDI functional skills**			
Ipsilateral CST	62.1 (59.7, 64.6)	66.5 (64.5, 68.5)	66.7 (64.7, 68.7)
Bilateral CST	68.7 (62.6, 74.7)	70.2 (66.2, 74.2)	69.6 (66.7, 72.5)
Contralateral CST	63.3 (57.6, 69)	64.7 (60.7, 68.6)	67.3 (64.3, 70.3)
**PEDI caregiver assistance**			
Ipsilateral CST	32.9 (31, 34.7)	35.2 (33.8, 36.7)	35.6 (34, 37.3)
Bilateral CST	36.2 (30.4, 42)	37.4 (35.2, 39.6)	38 (35.1, 41)
Contralateral CST	37.3 (26.5, 48.1)	37.3 (27.8, 46.8)	35.3 (30.6, 40)
**ABILHAND-Kids**			
Ipsilateral CST	1.6 (1.2, 2)	2.3 (1.9, 2.7)	2.4 (1.9, 2.8)
Bilateral CST	2.2 (1.2, 3.3)	2.7 (2.1, 3.3)	2.9 (1.7, 4.1)
Contralateral CST	2.3 (1.7, 2.8)	2.1 (1.9, 2.2)	3.3 (2.8, 3.8)

*CST, cortical spinal tract laterality; AHA, Assisting Hand Assessment; JTTHF, Jebsen-Taylor Test of Hand Function; COPM, Canadian Occupational Performance Measure; BBT, Box and Block Test; PEDI, Pediatric Evaluation of Disability Inventory*.

### Unimanual Dexterity

**Figure 3:**
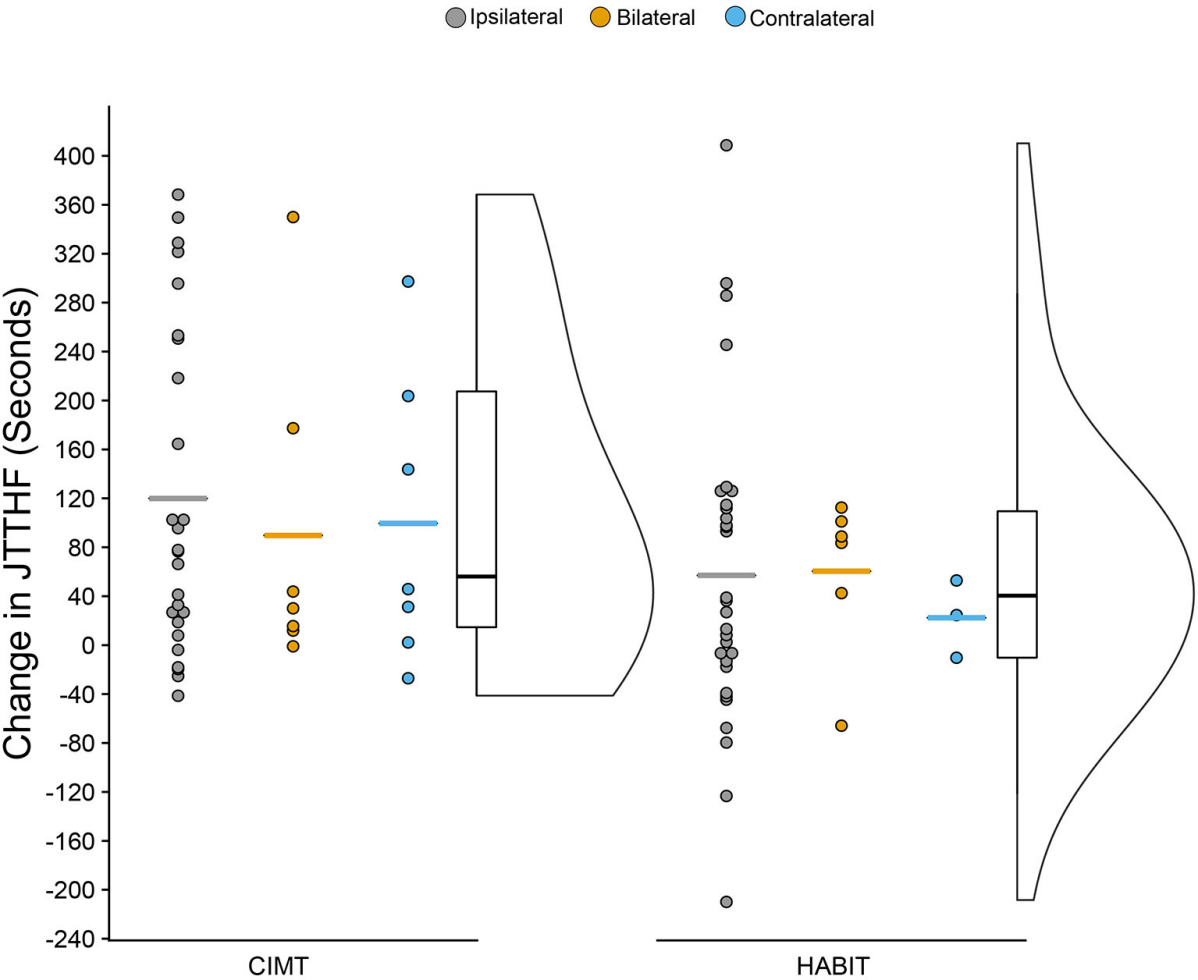
Raincloud Plot of Changes (Pre-test to Post-test) in JTTHF by Therapy Group and CST Connectivity (positive scores = improvements).

Figure 3 shows the change scores for the JTTHF for the more affected hand as a function of treatment and CST organization (mean scores can be seen in Table 2). There was a 111.1 s (24%) and a 56.3 s (11%) decrease in time for CIMT and HABIT, respectively {[*F*_(2, 72)_ = 44.0 *p* < 0.001, partial eta^2^ = 0.34], Table 2 and Figure 3}. Newman-Keuls *post-hoc* tests revealed a significant improvement between the pre-test and immediate post-test that was maintained at 6 months. There were no interactions between intervention group and test session or intervention group, CST connectivity pattern and test session (*p* > 0.05). There was no change in the less affected hand (*p* > 0.05).

There was an increase in the Box and Blocks performed with the more affected hand for both groups (12.29 for CIMT, 10.47 for HABIT) [*F*_(2, 72)_ = 50.77, *p* < 0.001 partial eta^2^ = 0.53] (Table 2). Newman-Keuls *post-hoc* tests revealed a significant improvement between the pre-test and immediate post-test that was maintained at 6 months. There were no interactions between intervention group and test session or intervention group, CST connectivity pattern and test session (*p* > 0.05). There was no change in the less affected hand (*p* > 0.05).

### Hand Use in Daily Functioning

For the ABILHAND-Kids (Table 2) both treatments resulted in significant improvement [*F*_(2, 72)_ = 1139.8 *p* < 0.001, partial eta^2^ = 0.97]. There were no interactions between intervention group and test session or intervention group, CST connectivity pattern and test session (*p* > 0.05).

Most goals defined in the COPM were bimanual (remaining ones were unimanual with the more affected hand). Most of the goals comprised self-care activities (e.g., dressing, grooming, and eating), followed by play (e.g., ball activities). Both groups had significant improvements in the COPM after the intervention on performance [*F*_(2, 72)_ = 89.06, *p* < 0.001, partial eta^2^ = 0.61] and on satisfaction [*F*_(2, 72)_ = 1139.9, *p* < 0.001, partial eta^2^ = 0.96] (Table 2). Twenty-four participants in the CIMT group and 29 in the HABIT group reached the MCID of 2 or more points for COPM performance. Twenty-five participants in CIMT group and 30 in the HABIT group reached the MCID of 2 or more points for COPM satisfaction. There were no interactions between intervention group and test session or intervention group, CST connectivity pattern and test session (*p* > 0.05).

Finally, there was an overall improvement in the PEDI-Functional Skills and PEDI- Caregiver Assistance in self-care over time [*F*_(2, 72)_ = 4727.9, *p* < 0.001, partial eta^2^ = 0.99] (Table 2). There were no interactions between intervention group and test session or intervention group, CST connectivity pattern and test session (*p* > 0.05).

### Children With a Bilateral CST

**Figure 4:**
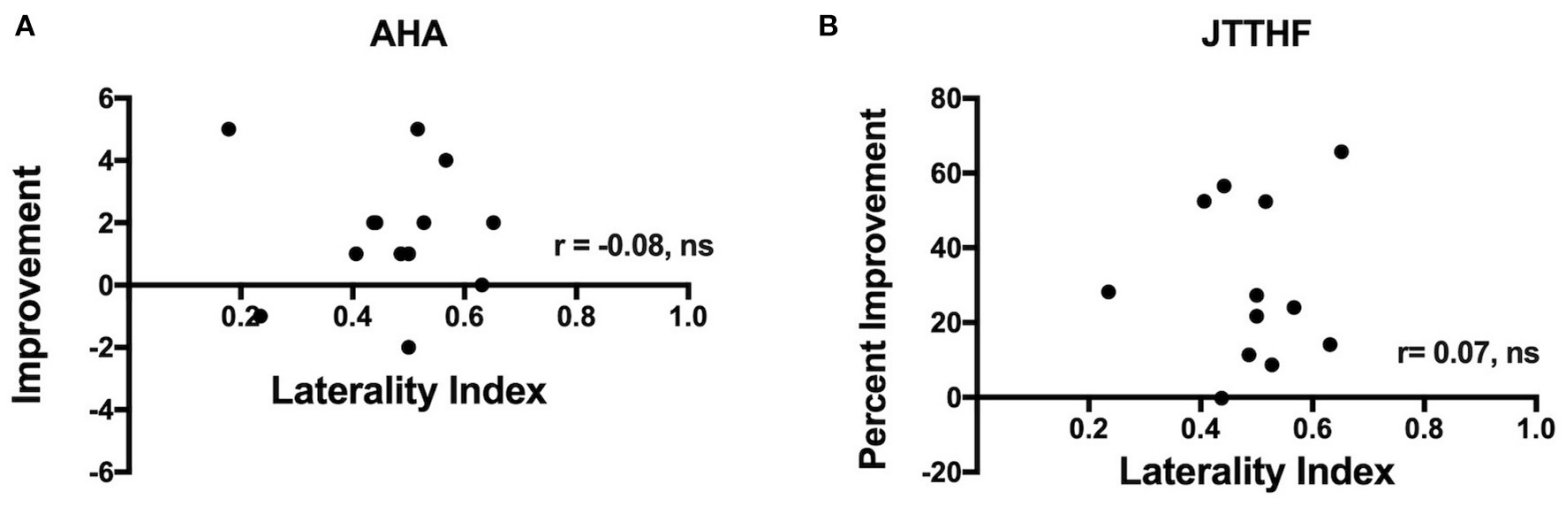
Plots showing correlations between laterality index and improvement in **(A)** AHA and **(B)** JTTHF immediately after the intervention. Note that one JTTHF change value was >3.5 SD from the mean and was excluded from the correlation.

Although the above analyses indicated that intervention efficacy was independent of CST laterality, we further examined children with a bilateral CST. We examined correlations between LI and the percent change in each outcome measure for the combined CIMT and HABIT groups (note that the groups were too small to considered by individual treatment). Figure 4 shows correlations between LI and changes in AHA (A) and JTTHF (B). As seen in the figure, there was no significant relation between change scores and LI for the AHA (*r* = −0.08) or JTTFF (*r* = 0.51, but *r* = 0.07 when an outlier >3.5 SD from the mean change score was removed). There were no significant correlations between LI and change in any other outcome measure (BBT: *r* = 0.09, pCOPM-Performance *r* = −0.23, COPM-Satisfaction *r* = 0.24, ABILHAND: *r* = −0.36, PEDI-Caregiver Assistance: *r* = −0.34, PEDI-Functional Skills: *r* = 0.14).

Given the relatively small number of participants with purely CST connectivity, we also reanalyzed all measures considering just two groups: contralateral CST connectivity present (combining the contralateral and bilateral groups) or contralateral CST connectivity absent (ipsilateral group). There still were no interactions between CST connectivity group and treatment for any measure.

### Predictors of Improvement

There were no statistically significant predictors of improvement among the many potential covariates examined. Specifically, there was no significant association between MACS level (*p* > 0.1), lesion type (*p* > 0.2), sex (*p* > 0.6), age (*p* > 0.4), side of lesion (*p* > 0.4), and baseline function (*p* > 0.1) on improvement in any outcome measure.

## Discussion

This is the first study, to our knowledge, to prospectively examine how CST laterality in children with USCP might mediate functional recovery following intensive unimanual or bimanual therapy. We found the efficacy of intensive training to be independent of CST connectivity pattern for all measures. This finding did not support our hypothesis that improvements in UE function following either CIMT or bimanual training depend on CST laterality and type of training (unimanual vs. bimanual) in children with USCP. That is, children with ipsilateral, bilateral, and contralateral CST connectivity improved equally in both CIMT and HABIT. We discuss possible reasons for these findings.

### CST Connectivity Does Not Predict Treatment Efficacy

When brain injury is superimposed on development, the motor system exhibits an extraordinary capacity to adapt. In children with USCP, this flexibility is manifested as rewiring of the CST and has intricate consequences for sensorimotor function. Studies suggest CST laterality is associated with the magnitude of UE impairments (10, 17–19). Yet, the variability in UE function observed in children with USCP and the observation that an ipsilateral CST may provide a neural substrate for plasticity (21, 22) has made a precise understanding of how developmental reorganization impacts motor skills difficult. Perhaps more importantly, there has been contradictory evidence regarding the role CST laterality may play in mediating response to therapy. Studies of CIMT have proposed ipsilateral CST connectivity as a limiting factor to recovery (16, 31), although the relationship between CST connectivity and CIMT outcomes is not unequivocal (8). It should be noted that both of these studies had small sample sizes (*n* = 16 in each study). In a larger study of HABIT (*n* = 33) the results suggest children improve regardless of connectivity pattern (21). We sought to adjudicate between these differences in a larger, prospective randomized control trial.

Despite variability in outcomes, overall children with ipsilateral, contralateral, and bilateral CST connectivity patterns improved on all outcome measures for both CIMT and HABIT. Although we recruited only a small sample of children with contralateral CST connectivity, their responsiveness to CIMT and HABIT is consistent with previous studies. Combining this group with children with bilateral connectivity did not change this finding, and the strength of the contralateral projections in the bilateral CST group did not relate to the outcomes. The discrepancy in improvements following CIMT for the children with ipsilateral CST connectivity seen in our study and that of Islam et al. (8) and not in the study by Kuhnke et al. (16) potentially may be explained by several factors. First the participants in the Kuhnke et al. (16) study were considerably older, with a mean age of 17 years (range 10–30 years) compared to our study with a mean age of 9.5. Thus, it is possible that decreased neuroplasticity or the long development of compensatory strategies and life and treatment experiences may have limited the response to treatment in some individuals or that there may be an interaction between age and CST connectivity. However, we did not find a relation between age and outcomes for any measure in the present study. Furthermore, the study by Islam et al. (8) also had an older age group (age 10–16 years) yet reported improvements in participants with ipsilateral connectivity. Thus, this may not be the primary reason for the discrepancy.

The discrepancy in findings may be due to the differing outcomes. Our study and that of Islam et al. (8) used the AHA and JTTHF to determine efficacy of assisting hand use in bimanual activities and unimanual dexterity, respectively. These measures are validated in these age groups. The Kuhnke et al. (16) study used the Wolf Motor Function Test (WMFT) to determine unimanual dexterity, which is validated for adults who had experienced a stroke. As acknowledged by the authors, this test may be appropriate for a large number of their participants who were within the adult age range, but is not validated for the younger participants in their study. The outcomes may be test-dependent, as evidenced by the lack of changes on the Melbourne Assessment reported by Islam et al. (8). Nonetheless, there is some overlap in the manual activities between the WMFT and JTTHF, so it is unclear whether the findings may be due to the differing tests.

The differences between studies could also be due to baseline severity of hand impairments. There is not agreement across studies regarding the effects of severity and outcome following intensive treatment. For example, in a small study of CIMT we found children with greater severity improved more following CIMT (24). Poorer baseline hand function predicted a best response for unimanual capacity immediately after CIMT or bimanual training (9). Similarly, Simon-Martinez et al. (53) found better improvements among children with worse bimanual hand function following combined CIMT and action observation. However, we did not find a relationship between severity and outcomes following larger studies of CIMT and HABIT (2, 4, 10). It should be noted that the lack of overlapping measures precludes us from determining severity differences between our study and that of Kuhnke et al. (16). However, an important consideration is that most studies have exclusion criteria that don't allow the children with the mildest or most severe hand function participate, and thus the relationship cannot clearly be determined across the USCP population. Given the large heterogeneity of individuals with USCP, the discrepancies in outcomes may be due to the specific sample enrolled. To our knowledge, our sample of children with ipsilateral CST connectivity participating in CIMT and bimanual training is the largest to date, and this large sample may suggest that even children with ipsilateral connectivity benefit following either CIMT or bimanual training.

### Response to Treatment

Despite significant changes on the AHA for both the HABIT and CIMT groups, the majority of participants did not reach the SDD. We did not find any factors that relate to the change in AHA scores. It is possible that since 70% of our sample had participated in our prior CIMT or HABIT (*n* = 23) studies or had received varying forms of CIMT (*n* = 35) ranging from wearing a sock in usual and customary care to full programs with a cast worn 24/7 at other sites prior to participating, there may have been a ceiling effect. Analysis of the children who did and did not receive previous intensive treatment suggested similar gains, although the latter group was quite small. Interestingly, 3 of the 5 participants in the CIMT group who reached the SDD for the AHA (Figure 2) had a contralateral CST pattern. Thus, with a larger number of these individuals the findings might suggest they respond better on average than children with other CST connectivity patterns. Nonetheless, children with ipsilateral CST connectivity patterns also improved and were among the children who reached the SDD for CIMT, and were the most common CST subgroup reaching the SDD for HABIT.

Despite the small individual AHA changes, significant changes were found across groups for all measures. More than two-thirds of the participants across both groups exceeded the MCID for goal performance as rated by caregivers. Thus, the majority of parents perceived clinically meaningful improvements in functional goals related to hand use.

Our finding that children improve equally across CST connectivity groups in both interventions is consistent with a systematic review that concluded that the minimum threshold dose needed to elicit improvements in children with USCP is 30–40 h (54) and at least 60 h for optimal improvements (55). Although the studies reviewed did not stratify by CST connectivity, our interventions involved ~3 × the minimum dose required to elicit changes and 50% more than the recommended intensity. Moreover, our motor learning-based, task-specific training, which also included functional goal training, are aligned with the type of interventions that lead to efficacy at a lower dose than general for UE motor training (54). Animal models suggest that high intensity training can result in increases in synaptic density in M1 (56), and increase cholinergic spinal interneurons (57). However, the high dose of treatment in the present study may have contributed to the lack of treatment differences based on CST connectivity. It is conceivable that differences (especially for the ipsilateral CST group) would be observed at lower doses.

### Other Neurological Predictors of Efficacy

Although CST connectivity patterns seemingly do not predict treatment outcome, there may be other brain areas that are more predictive. For example, children with greater structural, functional and connective brain damage have been shown to exhibit enhanced responses to bimanual intervention (58).

Functional connectivity of sensorimotor networks may differ depending on patterns of CST reorganization. Simon-Martinez et al. (59) found that children with a contralateral CST show increased connectivity between M1 and pre-motor cortices, whereas children with a bilateral CST show higher connectivity between M1 and somatosensory association areas. Impaired sensation (60, 61) and sensorimotor connectivity (62, 63) is related to poor motor performance. However, children with poor sensory function had larger improvements following CIMT or CIMT plus action observation (53). Thus, the integrity of the sensory tracts and whether they are maintained in the lesioned hemisphere (33, 64) may relate to functional improvements. Furthermore, brain lesion type and resulting volumetric changes (65) and the integrity of the corpus callosum have also been shown to relate to hand function (66). It is not directly known whether integrity of these interhemispheric connections is predictive of treatment efficacy. It is likely that there are complex interactions between the integrity of various areas and treatment outcome that are beyond the scope of the present study.

### Limitations

Despite being one of the largest studies comparing CIMT with bimanual training, there were not an equal number of participants with each CST connectivity pattern. Although we did have a large number of participants with ipsilateral connectivity, adding to the confidence that such connectivity is not maladaptive, we had a small number of children with purely a contralateral pattern. This may be because these children may have very mild hand function impairments in which they do not qualify or whose parents do not view the effort/potential benefit as being attractive. However, the responsiveness of these individuals across studies is not in doubt, and the findings held true even when the contralateral and bilateral groups were combined. The latter group is perhaps more complex. It is unclear whether they can be considered a homogeneous group given the laterality indices varied considerably across participants. Nonetheless, these indices did not correlate with outcomes, and further study is warranted.

The large number of participants who had received intensive therapies prior to participating in this study may have limited our gains in UE function by creating ceiling effects. Although the gains were similar whether or not children had received previous intensive therapy, the favorable response to prior therapy may have influenced the decision to participate in this study, and thus these could potentially be “best responders.” The small number of first-time participants precludes us from examining whether CST connectivity predicts treatment outcomes in first-time participants.

## Conclusions

The present study suggests children with ipsilateral, bilateral, and contralateral CST connectivity improved equally in both CIMT and HABIT. Thus, children with an ipsilateral CST, previously thought to be maladaptive, have the capacity to improve as well as children with a contralateral or bilateral CST following intensive CIMT or Bimanual training.

## Data Availability Statement

The raw data supporting the conclusions of this article will be made available by the authors, without undue reservation.

## Ethics Statement

The studies involving human participants were reviewed and approved by Institutional Review Boards of Teachers College, Columbia University, Burke Neurological Institute and Weill Cornell Medicine. Written informed consent to participate in this study was provided by the participants' legal guardian/next of kin.

## Author Contributions

KF and AG conceptualized the project and oversaw its implementation. KF, AG, CF, and MB wrote the manuscript. KF, CF, KC, H-CK, VF, MR, AS, and TC performed TMS and contributed to analyses. CF, MB, Y-CH, VF, H-CK, YB, and KC contributed to clinical supervision of treatment. JC assessed neurological reports and oversaw TMS safety. All authors provided editorial input to the final manuscript.

## Conflict of Interest

The authors declare that the research was conducted in the absence of any commercial or financial relationships that could be construed as a potential conflict of interest.
